# Evaluation of salivary cortisol levels in peri-implant diseases: An analytical observational study

**DOI:** 10.34172/japid.025.3817

**Published:** 2025-10-19

**Authors:** Melika Sadrejamali, Saba Mohammadi, Nasrin Esfahanizadeh

**Affiliations:** ^1^Faculty of Health, Deakin University, Melbourne, Australia; ^2^Periodontics Department, University of Pennsylvania, Philadelphia, Pennsylvania, USA; ^3^Periodontics Department, Islamic Azad University Dental Branch of Tehran, Tehran, Iran

**Keywords:** Dental implants, Hydrocortisone, Mucositis, Peri-implantitis, Saliva

## Abstract

**Background.:**

A well-documented positive correlation exists between salivary cortisol levels (SCLs) and periodontal disease. Given the clinical and pathophysiological similarities between peri-implant diseases and periodontal conditions, this study aimed to explore the association between SCLs and peri-implant mucositis and peri-implantitis.

**Methods.:**

An analytical observational study was conducted involving 86 patients who had been using dental prostheses for a minimum of one year. Unstimulated salivary samples were collected from all the participants. Clinical assessments included periodontal probing depth (PPD), papilla bleeding index (PBI), Mombelli modified plaque index (mPI), and radiographic evaluation of bone loss. Based on clinical and radiographic findings, the subjects were categorized into three groups: (1) individuals with healthy peri-implant tissues, (2) patients with peri-implant mucositis, and (3) patients with peri-implantitis. Salivary cortisol concentrations were quantified using an enzyme-linked immunosorbent assay (ELISA). Statistical analysis was conducted using one-way analysis of variance (ANOVA), followed by independent t-tests and post hoc Tukey comparisons.

**Results.:**

Significant differences were observed in mean PPD values between the three groups (*P*<0.05), with the peri-implantitis group exhibiting the highest values. Likewise, mPI scores varied significantly across the groups (*P*<0.05). However, no significant differences were detected in SCLs between the three groups.

**Conclusion.:**

Within the limitations of this study, no significant association was identified between SCLs and peri-implant disease. Further studies with larger sample sizes and longitudinal designs are recommended to validate these findings.

## Introduction

 Over the past four decades, implant treatment has significantly revolutionized modern dentistry and is now regarded as one of the most predictable and effective modalities for replacing missing teeth.^1^ Despite its high success rates, dental implant treatment is not devoid of complications. In recent years, a growing prevalence of peri-implant inflammatory conditions has been reported, which pose significant challenges to long-term implant success. Peri-implant diseases are characterized by nonspecific inflammatory responses in the peri-implant soft and hard tissues, clinically classified as peri-implant mucositis and peri-implantitis.^1^ The primary etiologic factor in the development of these conditions is the accumulation of microbial biofilm on the implant surface. However, several additional risk factors, including smoking, a history of periodontitis, genetic susceptibility, systemic diseases such as diabetes mellitus, and inadequate oral hygiene, may exacerbate the host’s inflammatory response and contribute to disease progression.^2^

 Peri-implant mucositis is defined as a reversible inflammatory condition confined to the soft tissues surrounding a dental implant, without radiographic evidence of supporting bone loss. Clinically, it is characterized by bleeding on probing (BoP) and may be accompanied by erythema, edema, and, in some cases, suppuration. Substantial evidence supports the role of dental plaque as the principal etiologic factor in the development of peri-implant mucositis, reflecting the pathogenesis of gingivitis in natural dentition. If not adequately managed, both gingivitis and peri-implant mucositis may progress to periodontitis and peri-implantitis, respectively, resulting in irreversible tissue destruction and potential implant failure.^2^

 The transition from peri-implant mucositis to peri-implantitis closely parallels the progression of gingivitis to periodontitis; however, it is associated with distinct clinical, immunological, and microbiological profiles.^3^ In contrast, peri-implantitis is an irreversible, advanced pathological condition associated with microbial plaque accumulation, and it is primarily driven by the same bacterial species implicated in periodontitis. Unlike peri-implant mucositis, peri-implantitis involves progressive loss of supporting bone and is considered a more severe manifestation of peri-implant disease. There is currently no single definitive diagnostic criterion for peri-implantitis; however, its diagnosis is based on a combination of clinical and radiographic findings. Hallmark features include signs of inflammation such as BoP, suppuration, increased probing depth (PD), mucosal recession, and radiographic evidence of peri-implant bone loss relative to previous baseline assessments.^2,4^

 Psychological stress, tobacco use, inadequate oral hygiene, diabetes mellitus, and genetic predisposition are well-established risk factors that contribute to the development and progression of both periodontal and peri-implant diseases.^5^ Psychological stress may influence the periodontium through multiple biological pathways, including dysregulation of immune responses, alterations in microbial biofilm composition, impaired collagen metabolism and protein turnover, and the exacerbation of both systemic and local inflammatory processes.^6-8^ Stress can adversely impact periodontal health both directly and indirectly. Indirectly, it may lead to poor oral hygiene, increased smoking and alcohol consumption, and unhealthy dietary habits. Directly, stress alters salivary composition, reduces gingival blood flow, and modulates immune responses, thereby promoting periodontal disease progression.^9^

 Cortisol, the principal glucocorticoid hormone with anti-inflammatory properties, is released into the bloodstream in both free and protein-bound forms. It is widely recognized as a biomarker of psychological stress and related psychiatric disorders, with circulating cortisol levels correlating directly with the intensity of stress experienced by an individual.^10^ Physiologically, cortisol modulates immune and inflammatory responses as well as tissue repair mechanisms, including those affecting the periodontium. These effects contribute to the onset and severity of periodontal diseases. Hingorjo et al^11^ demonstrated that patients with periodontitis exhibited significantly higher salivary cortisol levels (SCLs) and stress scores compared to healthy controls, suggesting a strong correlation. Furthermore, their study reported elevated clinical indicators, including PD, clinical attachment loss (CAL), and gingival index (GI) in the periodontitis group, reinforcing the association between elevated cortisol levels and increased periodontal disease severity. Similarly, La Monaca et al^10^ identified cortisol as a potential biomarker with predictive value for both periodontal and peri-implant diseases. However, they noted that cortisol levels may be influenced by systemic conditions such as anxiety and chronic hyperglycemia. Furthermore, Chang et al^12^ reported a linear correlation between SCLs and periodontal probing depth (PPD), independent of glycemic status, and emphasized depression as a significant psychological factor contributing to periodontal disease severity. These findings suggest that cortisol may serve as a valuable biomarker for peri-implant diseases; however, its predictive accuracy can be confounded by factors such as anxiety, tobacco use, and chronic hyperglycemia. However, some studies have not found any relationship between cortisol levels and periodontal status.^13,14^

 Alresayes et al^15^reported that cortisol concentrations in the peri-implant sulcular fluid (PISF) were significantly higher in patients with peri-implantitis compared to those without the condition. Similarly, Soysal et al^5^ observed that while psychological stress alone may not directly induce peri-implantitis in otherwise healthy individuals, it can enhance susceptibility to inflammation by modulating cytokine expression. Additionally, Ali et al^16^ found elevated PISF cortisol levels in both type 2 diabetic and non-diabetic individuals with peri-implantitis, further underscoring the association between stress biomarkers and peri-implant disease severity.

 Integrating cortisol assessment into routine dental evaluations may facilitate the development of personalized treatment strategies that address both biological and psychological factors contributing to peri-implant diseases. Given the information gap regarding the relationship between cortisol levels and inflammatory conditions around dental implants, this research examined SCLs in peri-implant mucositis and peri-implantitis cases.

## Methods

###  Patients

 This analytical observational study employed a non-random convenience sampling method to recruit participants from individuals referred to the Dental Implant Department of the Faculty of Dentistry. Ethical approval was obtained from the Ethics Committee of the Faculty of Dentistry at the Islamic Azad University of Medical Sciences (Ethics Code: IR.IAU.DENTAL.REC.1399.259). All the procedures were carried out in accordance with the ethical principles outlined in the Declaration of Helsinki. Furthermore, the study was conducted in compliance with the STROBE (Strengthening the Reporting of Observational Studies in Epidemiology) guidelines.

 The participants were eligible for inclusion if they were ≥ 18 years old, had at least one screw-type dental implant with a rough surface, had completed a minimum of one year since implant placement, and were actively using their prostheses in functional occlusal loading.

 Exclusion criteria included: use of antibiotics or nonsteroidal anti-inflammatory drugs (NSAIDs) within the past three months; presence of implant mobility; pregnancy or lactation; history of autoimmune disorders, malignancy, cardiovascular diseases, or other acute systemic conditions; prior treatment for peri-implantitis or periodontitis within the last six months; uncontrolled diabetes mellitus (HbA1c > 7%); tobacco use; and current use of medications such as antihypertensives, immunosuppressants, corticosteroids, diuretics, drugs affecting salivary gland function (e.g., antihistamines and tricyclic antidepressants), or psychotropic agents (e.g., antidepressants, antipsychotics, anxiolytics, sedatives). Additional exclusion criteria included undergoing orthodontic therapy, active treatment for psychological stress, acute oral or systemic disease, pulpal pathology, oral infections, and any diagnosed psychiatric disorders.

###  Saliva sampling

 A single calibrated examiner conducted all clinical measurements to ensure consistency. Saliva sampling was performed before any clinical examination to prevent contamination from bleeding sites.^17^ To minimize bias in salivary cortisol assessment, several standardization protocols were implemented. Unstimulated whole saliva was collected using the passive drooling (spitting) method between 9:00 and 11:00 a.m., a time window chosen to reduce the influence of circadian variation. During collection, the participants were seated in a relaxed, upright position. They were instructed to abstain from eating, drinking, or tooth brushing for at least one hour before sampling and rinse their mouths with water immediately beforehand. Approximately 1 mL of saliva was collected from each participant and stored in sterile microtubes at −20 °C until analysis.^14^ Free salivary cortisol concentrations were quantified using a commercially available enzyme-linked immunosorbent assay (ELISA) kit (ZellBio Human Salivary Cortisol ELISA Kit), which has a reported sensitivity of 1 ng/mL. Cortisol levels were expressed in ng/mL and recorded in the patients’ clinical files. The established reference range for SCLs was 2.5–10 ng/mL.^18^

###  Examining clinical indices

 Comprehensive oral and periodontal assessments were conducted to evaluate the peri-implant status of all the participants. Clinical parameters included PPD, papilla bleeding index (PBI), Mombelli modified plaque index (mPI), and radiographic evaluation of marginal bone loss. These indices were used to determine the peri-implant condition of each subject. Based on clinical and radiographic findings, the patients were classified into one of three diagnostic groups: (1) healthy peri-implant state, (2) peri-implant mucositis, or (3) peri-implantitis.

 PPD, defined as the distance from the gingival margin to the base of the gingival sulcus, was measured at four sites around each implant (distobuccal, buccal, mesiobuccal, and lingual) using a plastic periodontal probe with an 0.5-mm tip diameter and a gentle standardized probing force. Measurements were recorded in millimeters.^19^

 The mPI was assessed at four sites per implant: mesiobuccal, distobuccal, mesiolingual, and distolingual.^20^ BOP was evaluated 30 seconds after probing using the PBI developed by Saxer and Mühlemann.^21^ A periodontal probe was gently inserted into the sulcus at the base of the mesial papilla and moved coronally toward the papillary tip, then repeated for the distal papilla. The presence of bleeding was noted.^22^ The extent of marginal bone loss was evaluated radiographically using a parallel periapical digital technique. Bone loss was determined by measuring the vertical distance from the implant platform level (IPL) to the most apical point of bone-to-implant contact.^21,23^

 The patients were categorized into clinical groups according to established diagnostic criteria. The peri-implant mucositis group comprised individuals presenting with BOP, peri-implant edema, or suppuration, with radiographic bone loss of < 2 mm. The peri-implantitis group included patients exhibiting BOP and/or pus discharge from at least one implant surface within 60 seconds of probing, a periodontal probing depth (PPD) of ≥ 4 mm, and marginal bone loss of ≥ 2 mm. The participants were classified as healthy if they exhibited no BOP or bleeding limited to a single surface attributed to probing trauma, no signs of pus discharge, and peri-implant bone loss of < 0.2 mm.^24^

###  Salivary cortisol analysis via ELISA

 All reagents, including standard and control solutions supplied with the ELISA kit, were gently agitated before use to ensure homogeneity and temperature equilibrium. The salivary samples were centrifuged at 3000 rpm (approximately 2600 × g) for 15 minutes at 4 °C to remove cellular debris. Subsequently, 50 μL of each patient sample, along with standards and controls, was dispensed into the designated wells of the ELISA plate.

 Next, 100 μL of conjugate solution was added to each well. The plate was incubated at room temperature for 45 minutes to allow for antigen‒antibody binding. Following incubation, the wells were washed three times with 300 μL of the provided wash buffer using an automated ELISA washer to eliminate unbound substances.

 Thereafter, 150 μL of substrate solution was added to each well to initiate color development through enzymatic reaction with the conjugate. After 20 minutes of incubation, 50 μL of stop solution was added to terminate the reaction. Absorbance was immediately measured at 450 nm using an ELISA microplate reader.^25^

###  Sample size determination

 The sample size was determined based on data from the study by Jabali et al,^26^ which investigated SCLs. Assuming a standard deviation of 2.5 ng/mL and a minimum detectable difference of 2.5 units between groups, with a significance level (α) of 0.05 and a statistical power of 80%, a minimum of 17 participants per group was required. To accommodate multivariate analysis involving at least three groups and ensure adequate statistical power, 45 participants were deemed necessary.

###  Data analysis

 Descriptive statistics, including the mean and standard deviation of each clinical index, were calculated for both treatment and control groups using IBM SPSS Statistics Version 20. To compare SCLs among the three study groups, a one-way analysis of variance (ANOVA) was conducted, followed by post hoc Tukey tests for pairwise comparisons. Additionally, independent-samples t-tests were applied to two-group datasets where applicable.

## Results

 A total of 800 patients from the Implant Department were initially contacted by phone. Of these, approximately 130 attended an in-person screening, and 86 individuals met the study’s inclusion criteria. Among the enrolled participants, 29 were classified as having healthy peri-implant tissues, 31 as having peri-implant mucositis, and 26 as having peri-implantitis. The mean age of the sample was 51.63 ± 12.5 years, comprising 24 males and 62 females.

 ANOVA indicated no significant difference in mean SCL between the three diagnostic groups (*P* > 0.05, Table 1). Further pairwise comparisons using post hoc Tukey tests also revealed no significant differences in mean SCL between any of the groups (*P* > 0.05; Table 2).

 One-way ANOVA revealed a significant difference in the mean PPD between the three study groups (*P* < 0.05, Table 3). Subsequent post hoc analyses using the Tukey tests showed that the mean PPD was significantly greater in patients with peri-implantitis compared to those with peri-implant mucositis (*P* < 0.05), and significantly higher when compared to healthy individuals (*P* < 0.05). However, the difference in mean PPD between healthy participants and those with peri-implant mucositis was not significant (*P* > 0.05, Table 4).

 The paired independent-samples t-test revealed no significant difference in the mean PBI between the two experimental groups (*P* = 0.11). In contrast, one-way ANOVA revealed highly significant differences in the mean mPI between the three groups (*P* < 0.001, Table 5).

 Pairwise comparisons using post hoc Tukey tests (Table 6) confirmed that the differences in mean mPI values between all three groups were significant (*P* < 0.05), indicating distinct levels of plaque accumulation associated with peri-implant health status.

**Table 1:** Mean difference ( ± standard deviation) of salivary cortisol (ng/mL) levels between the three experimental groups

**Experimental group**	**Number**	**Mean±SD**	**Minimum**	**Maximum**	**F-statistic**	***P*** ** value**
Peri-implantitis	26	11.27 ± 4.50	1.91	18.27	1.84	0.16 ^a^
Peri-implant Mucositis	31	9.61 ± 3.17	0.10	13.36		
Healthy	29	11.37 ± 4.24	5.86	20.95		
Total	86	10.71 ± 4.01	0.10	20.95		

^a)^ No statistically significant differences between groups (*P* > 0.05).

**Table 2:** Mean difference ( ± standard error) of salivary cortisol (ng/mL) levels in paired group comparisons

**Comparison of groups**		**Mean±SE**	***P*** ** value**
Peri-implantitis	Peri-implant mucositis	1.66 ± 1.05	*P* > 0.05^a^
	Healthy	-0.09 ± 1.07	
Peri-implant Mucositis	Peri-implantitis	-1.66 ± 1.05	
	Healthy	-1.75 ± 1.02	
Healthy	Peri-implantitis	0.09 ± 1.07	
	Peri-implant mucositis	1.75 ± 1.02	

^a^No statistically significant differences between groups.

**Table 3:** Mean difference ( ± standard deviation) in PPD (mm) between the three experimental groups

**Experimental group**	**Number**	**Mean±SD**	**Minimum**	**Maximum**	**F-statistic**	***P*** ** value**
Peri-implantitis	26	4.55 ± 0.56	4	6.25	16.47	0.0001^a^
Peri-implant Mucositis	31	3.06 ± 0.82	1.67	5.25		
Healthy	29	2.71 ± 0.62	1.75	4.00		
Total	86	3.22 ± 0.97	1.67	6.25		

^a^ Statistically significant differences between groups (*P* < 0.001).

**Table 4:** The mean difference ( ± standard error) of the PPD (mm) in the paired comparison of groups

**Comparison of groups**		**Mean±SE**	***P*** ** value**
Peri-implantitis	Peri-implant mucositis	0.90 ± 0.22	0.0001^a^
	Healthy	1.26 ± 0.22	0.0001^a^
Peri-implant Mucositis	Peri-implantitis	-0.90 ± 0.22	0.0001^a^
	Healthy	0.35 ± 0.21	0.234
Healthy	Peri-implantitis	-1.26 ± 0.22	0.0001^a^
	Peri-implant mucositis	-0.35 ± 0.21	0.234

^a^ Statistically significant differences between groups (*P* < 0.001).

**Table 5:** The mean difference ( ± standard deviation) of the mPI in the three experimental groups

**Experimental group**	**Number**	**Mean±SD**	**Minimum**	**Maximum**	**F-statistic**	***P*** ** value**
Peri-implantitis	26	1.26 ± 0.43	0.75	2.40	24.32	< 0.001^a^
Peri-implant mucositis	31	0.94 ± 0.33	0.06	1.50		
Healthy	29	0.46 ± 0.50	0.00	1.50		
Total	86	0.88 ± 0.53	0.00	2.40		

^a^ Statistically significant differences between groups (*P* < 0.001).

**Table 6:** The mean difference ( ± standard error) of mPI in the paired comparison of groups

**Comparison of groups**		**Mean±SE**	***P*** ** value**
Peri-implantitis	Peri-implant mucositis	-0.06 ± 0.14	> 0.05^a^
	Healthy	-0.20 ± 0.15	
Mucositis	Peri-implantitis	0.06 ± 0.14	
	Healthy	-0.13 ± 0.14	
Healthy	Peri-implantitis	0.20 ± 0.15	
	Peri-implant mucositis	0.13 ± 0.14	

^a^No statistically significant differences between groups.

## Discussion

 The relationship between psychological stress and oral diseases was first reported in the 1970s, when psychosocial stress was linked to an increased incidence of virus-induced mucosal lesions, such as those caused by rhinovirus and coxsackievirus.^23^ Since then, considerable research has been devoted to elucidating the molecular mechanisms through which stress influences inflammatory conditions of the oral cavity, including periodontal and peri-implant diseases.^27^

 Stress induces systemic and local alterations in immune function via an intricate network of neuroendocrine-immune interactions. It affects the balance between T-helper cell subtypes (Th1/Th2). It has been associated with elevated secretion of pro-inflammatory cytokines such as interleukin-6 (IL-6), which may play a critical role in the pathogenesis and progression of periodontal disease.^27^

 For more than five decades, cortisol has been recognized as a precise, reliable, and non-invasive biomarker for assessing chronic stress in both pediatric and adult populations.^28^ In addition to its diagnostic utility, cortisol exerts significant immunomodulatory effects. It can suppress the immune cascade while concurrently promoting the production of inflammatory cytokines.^29^ Among these, interleukin-1β (IL-1β), a key proinflammatory cytokine, is found in elevated concentrations in unstimulated saliva of individuals with periodontitis and peri-implant diseases.^30^

 Cortisol also downregulates T-cell-mediated immune responses, promoting a shift toward a humoral (Th2-mediated) immune profile. This immunological imbalance facilitates the proliferation of microbial species that further stimulate cellular immune responses, thereby contributing to the chronic inflammatory milieu associated with periodontal and peri-implant pathology.^31^

 Cortisol compromises host defense mechanisms against periodontal pathogens by inhibiting the production of secretory immunoglobulins and reducing neutrophil function. These immunosuppressive effects facilitate microbial persistence, promote inflammatory responses, and contribute to tissue degradation within the periodontium, ultimately playing a significant role in the initiation and progression of periodontal disease.^32^ Furthermore, periodontal tissues express glucocorticoid receptors that are responsive to cortisol released via the hypothalamic-pituitary-adrenal (HPA) axis. Notably, keratinocytes in the oral mucosa respond directly to adrenocorticotropic hormone (ACTH) and can synthesize cortisol endogenously.^33^ This local hormone production may further influence inflammatory processes in periodontal tissues.

 Current evidence suggests that elevated SCL may be a risk factor for periodontal diseases. Research has demonstrated a correlation between cortisol concentrations in saliva and gingival crevicular fluid (GCF) among individuals with periodontitis, indicating that affected individuals exhibit elevated cortisol levels in both fluids compared to healthy controls. Furthermore, studies have shown that psychological factors such as anxiety and depression can significantly influence cortisol levels in oral fluids, including saliva and GCF.^14,34^

 There are several methods for assessing cortisol levels in the body. While most of the cortisol in the bloodstream is protein-bound, only a small fraction exists in its “free,” biologically active form. SCL reflects this unbound fraction and thus serves as an accurate surrogate for free serum cortisol.^35^ Studies have indicated that blood cortisol measurements may yield misleading results due to the stress-induced response triggered by blood sampling. Consequently, non-invasive sampling methods such as urine, feces, and saliva are preferred.^36^ Among these, salivary cortisol assessment is considered superior, as it directly reflects the biologically active hormone, is unaffected by salivary flow rate, and rapidly equilibrates with serum cortisol.^13^ Furthermore, saliva collection is a painless and straightforward procedure that minimizes stress-related activation of the adrenal axis, unlike venipuncture.

 Saliva is a highly stable medium for cortisol analysis, with the hormone remaining stable at room temperature for up to seven days.^13,35^ The collection process is simple and does not require medical personnel; trained individuals can easily perform the procedure.^36^ These advantages make saliva an ideal biological fluid for cortisol measurement, which is why it was utilized in this study. The findings of this study demonstrated significant differences in the PPD and mPI clinical parameters among the three groups (*P* < 0.05). However, no statistically significant difference in mean SCL was observed between the peri-implant mucositis, peri-implantitis, and healthy groups (*P* > 0.05). Develioglu et al^17^ demonstrated that the severity of chronic periodontitis is positively associated with elevated SCL, whereas no such association was observed with other salivary stress markers. Their findings indicated that individuals with severe chronic periodontitis exhibited significantly higher salivary cortisol concentrations than those with milder forms of the disease. Similarly, Obulareddy et al^37^ investigated the relationship between SCL and periodontitis in patients with and without psychological stress. Their findings revealed that the mean SCL was highest in patients experiencing both periodontitis and stress, supporting the notion that salivary cortisol is positively correlated with both chronic periodontitis and psychological distress. In another study, Naghsh et al^14^ examined unstimulated SCLs in patients with and without chronic periodontitis. They found that both mean SCL and PD were significantly higher in individuals with periodontitis than in healthy controls.

 Additionally, Cakmak et al^38^ investigated the effect of nonsurgical periodontal treatment on stress hormone levels in GCF. Their findings indicated that, irrespective of disease severity, cortisol levels and all clinical parameters (CAL, PD, BoP, GI, and mPI) significantly decreased following treatment. In the present study, salivary testing was employed to assess cortisol levels, based on the findings of Johannsen et al,^39^ who reported that saliva testing offers greater accuracy than GCF in evaluating stress hormone concentrations.

 Although numerous studies have investigated the relationship between cortisol levels and periodontal disease, limited research has explored the association between cortisol and peri-implant diseases. Alresayes et al^15^ examined cortisol levels in PISF in individuals with and without peri-implantitis and reported significantly higher cortisol concentrations in those with the condition. In contrast, the present study assessed cortisol levels using saliva rather than PISF, which may account for the observed differences in findings between the two studies.

 Our study found no significant differences in SCL among patients with different peri-implant conditions, which contrasts with the findings of Ali et al,^16^ who reported elevated cortisol concentrations in the PISF of both type 2 diabetic and non-diabetic patients with peri-implantitis compared to healthy individuals. Ali et al^16^ also identified a significant correlation between PD and cortisol levels in non-diabetic peri-implantitis patients, suggesting that PISF cortisol may serve as a marker of local inflammation. The discrepancies between our results and those of Ali et al^16^ may be attributed to differences in the biological sample analyzed (saliva vs. PISF) and variations in patient populations. Additionally, Soysal et al^5^ reported that psychological stress may exacerbate peri-implant inflammation by modulating cytokine expression, specifically IL-1β, IL-6, and IL-10. This implies that stress-related mechanisms beyond cortisol, including pro-inflammatory cytokine pathways, may play a role in the pathogenesis of peri-implantitis. Together, the elevated PISF cortisol levels reported by Ali et al^16^ and the cytokine alterations described by Soysal et al^5^ underscore the potential involvement of localized regulatory mechanisms within the peri-implant environment, particularly in the presence of systemic conditions such as diabetes.

 La Monaca et al^10^ conducted a study on biomarkers in peri-implant crevicular fluid, identifying cortisol as one of the key indicators with predictive value for peri-implantitis, alongside IL-1β, VEGF, and sRANKL/OPG. However, they emphasized that the strength of evidence supporting cortisol’s predictive value is moderate, as its levels can be influenced by various factors, including anxiety, smoking, and chronic hyperglycemia. This variability in cortisol’s diagnostic reliability may help explain the discrepancies observed in the present study, where no significant differences in SCL were found between patients with different peri-implant conditions. Differences in sample types (saliva vs. PISF) and patient populations may also contribute to these inconsistencies.

 In this study, although SCLs were higher in patients with peri-implantitis compared to those with peri-implant mucositis and healthy individuals, the difference was not significant. This observation may suggest a potential positive association between SCL and the severity of peri-implant inflammatory disease, similar to the findings in periodontitis studies, such as that by Develioglu et al,^17^ which demonstrated a correlation between disease severity and cortisol concentration. In the present study, radiographic bone loss was used as the diagnostic criterion for peri-implantitis; however, the extent of bone loss was not quantitatively assessed, and no distinction was made between early and advanced stages of the disease. Therefore, future research should explore the relationship between SCL and varying degrees of peri-implantitis severity. It is plausible that a statistically significant association may emerge in more advanced cases, similar to findings in periodontitis, where markedly higher SCL have been observed in individuals with advanced or aggressive forms of the disease compared to those with mild periodontitis.^40^

 On the other hand, the use of whole saliva to measure cortisol may not adequately reflect localized peri-implant inflammation. Since whole saliva represents a pooled systemic response, it might not capture site-specific inflammatory activity as accurately as PISF. This is consistent with the findings of Haririan et al,^18^ who reported no significant differences in cortisol levels between healthy individuals and those with periodontal disease, whether measured in saliva or serum.

 The strengths of this study include its examination of the relationship between SCL and peri-implantitis, an area that has been less explored compared to periodontal tissues, where most studies have focused on cortisol levels and periodontitis. To the best of our knowledge, research similar to the present study has not yet been conducted. Furthermore, the large sample size (86 specimens) is another key strength of this study.

 One limitation of the present study is that psychological stress was assessed solely through biological markers (i.e., salivary cortisol) and did not include subjective measures. However, patients were asked whether they had experienced symptoms of anxiety or depression, or used medications associated with the treatment of these symptoms, in which case they were excluded from the study. Future research should consider incorporating validated stress assessment tools, such as the Perceived Stress Scale (PSS) or the Depression Anxiety Stress Scales (DASS), to complement biological findings and provide a more comprehensive understanding of the relationship between stress and peri-implant inflammatory conditions.

## Conclusion

 The results of this study indicated no significant difference in SCL between individuals with peri-implant mucositis or peri-implantitis and healthy individuals. However, further studies are necessary to evaluate the potential role of cortisol in diagnosing peri-implantitis and peri-implant mucositis.

## Competing Interests

 The authors declare that they have no competing interests regarding authorship and/or publications of this paper.

## Data Availability

 The datasets that were used in the present study have been deposited in the Harvard Dataverse under accession code https://doi.org/10.7910/DVN/VQHGPT.^41^

## Ethical Approval

 The study protocol was approved by the Ethics Committee of the Faculty of Dentistry at the University of Medical Sciences, with ethics code IR.IAU.DENTAL.REC.1399.259.
